# The Role of Information in Knowledge-How

**DOI:** 10.3389/fpsyg.2021.634968

**Published:** 2021-09-13

**Authors:** Jonathan Najenson, Nir Fresco

**Affiliations:** ^1^Department of Philosophy, Hebrew University of Jerusalem, Jerusalem, Israel; ^2^Department of Brain & Cognitive Sciences and Philosophy, Ben-Gurion University of the Negev, Beersheba, Israel

**Keywords:** knowledge, information, skill, motor control, automatization

## Abstract

Knowledge-how is the kind of knowledge implicated in skill employment and acquisition. Intellectualists claim that knowledge-how is a special type of propositional knowledge. Anti-intellectualists claim that knowledge-how is not propositional. We argue that both views face two open challenges. The first challenge pertains to the relationship between informational states and motor variability. The second pertains to the epistemic function of practice that leads to skill (and knowledge-how). The aim of this paper is to suggest a general conceptual framework based on functional information with both intellectualist and anti-intellectualist features. Our proposal, we argue, avoids the above challenges, and can further the debate on knowledge-how and skill.

## Introduction

An ongoing debate in epistemology concerns a kind of knowledge called “Know-How” (KH). This is the kind of knowledge an agent has when she possesses a skill, such as cycling or reading. According to intellectualists, KH is a species of the more familiar kind of knowledge: propositional knowledge (e.g., Stanley and Williamson, 2001; Stanley and Krakauer, 2013; Pavese, 2019). Anti-intellectualists deny that skills are exhausted by propositional knowledge, citing flexibility, context sensitivity, and the richness of motor representations as key reasons for rejecting the intellectualist view (Fridland, 2013; Christensen et al., 2016; Burnston, 2020).

We propose a hybrid view: the type of things we know when we know how to F is manifested through multiple informational relations, that can, but *need not*, be propositional. On our proposal, which builds on the conceptual framework of functional information (see, e.g., Fresco et al., 2018; Mann, 2018), practicing successful courses of action leads to the elimination of other possible courses of action, and to stabilization on some “optimal” course(s) of action. On this view, information is a triadic relation between a receiver, a difference-maker, and a state of affairs. From the receiver’s perspective, a difference-maker is informational due to the receiver having learned to respond to it in a regular and functional manner by altering the receiver’s internal state. To defend our proposal, we analyze the various ways in which different kinds of information play a role in guiding skillful actions and acquiring skills, suggesting that by gaining functional information individuals can adaptively modulate the variability of their movements and reduce the uncertainty about the outcome of their actions.

The paper proceeds as follows: section “The Debate in a Nutshell” gives a quick introduction to the debate between intellectualists and anti-intellectualists. Section “Informational States and Skilled Action” considers the first challenge faced by intellectualists and anti-intellectualists views: the relationship between informational states and motor variability. In section “The Importance of Practice for Knowledge-How,” we discuss the second challenge: the epistemic function of practice. Section “Two Desiderata for Accounts of KH” lays out two desiderata for an account of KH that can deal with these challenges. In section “A Conceptual Framework: Functional Information,” we sketch a proposal based on “functional information” for dealing with these challenges. Skillful agents make use of motor variability to flexibly adapt to variations in informational relations. Practice leads to gaining more functional information from an environmental cue, a signal, or even one’s own body, thereby reducing uncertainty about learned courses of action.

## The Debate in a Nutshell

Know-how is a peculiar phenomenon. It is a cognitive achievement, but much of what is associated with KH takes place at the sub-personal level. The epistemic profile of KH partly differs from the standard epistemic profile of propositional knowledge. The type of representation that could guide skillful performance appears to require peculiar features to guide action, such as flexibility to task demands alongside fast and automatic responsiveness. Which informational states constitute such a phenomenon?

According to intellectualists, propositional content constitutes KH (Stanley, 2011; Pavese, 2019). We take intellectualism to refer to a family of views that holds that KH is a subspecies of propositional knowledge. In these views, an agent counts as possessing the knowledge of how to F in virtue of having appropriate propositional states. Specifically, knowing how to F amounts to knowing that a course of action *w* is a way to F (e.g., Stanley, 2011). We will assume a version of this underlying thesis, according to which the mental states that are associated with KH—and, thus, are causally related (or at least supervene on the relevant causal process related) to the production of a skillful action—correspond with propositions.

On Stanley’s and Pavese’s versions of intellectualism, KH is a specific form of propositional knowledge, for it requires that one knows a proposition under a *practical mode of presentation*. We represent the world perspectivally, as determined by our representational abilities, such as our conceptual scheme. To guide skillful actions, one must represent the relevant proposition under a distinct perspective determined by our practical abilities, which yields a practical mode of presentation. Intended actions are represented in a particular way; “they represent a task as needing to be performed in accordance with a method, where a method breaks down the task in different ways depending on the system’s practical abilities” (Pavese, 2019, p. 798).

Those opposing intellectualism deny the thesis that KH is a subspecies of propositional knowledge.^1^ One reason for this is that skills are manifested through flexible goal-directed control and fine-grain sensitivity to the context of action (Sutton et al., 2011). Furthermore, flexible behavior may be manifested without being exclusively guided by personal-level representations (Levy, 2015). Such properties, anti-intellectualists argue, are not amenable to explanation in terms of propositional knowledge (Fridland, 2017b; Burnston, 2020).

In summary, according to intellectualism, KH is knowledge characterized by an agent’s standing in relation to a proposition under a practical mode of presentation. Anti-intellectualists deny that connection, claiming that propositional knowledge is not the right way to understand how skillful action is guided.

## Informational States and Skilled Action

We now turn to discuss the first challenge concerning how KH could potentially guide skillful actions. A central issue in this debate pertains to the nature of the informational states guiding and controlling a skillful action (Mylopoulos and Pacherie, 2017). Consider the multifarious nature of informational states implicated in skillful action. Agents can instruct and teach others how to accomplish a task. The information provided in instruction and teaching has to do with verbal understanding and action observation. In skillful actions, however, *motor* states are also implicated. These are states that represent features such as the kinematics of bodily movements. They are thought to reflect different neurocognitive mechanisms underlying distinct informational contents (Christensen et al., 2019).

Besides, skillful actions are compositional structures. Simpler and more isolated actions can be composed to form a more complex activity (Papineau, 2015). Playing basketball, for example, has action-components, such as throwing and catching the ball, running, passing, and blocking. Each one of these actions can be further decomposed. For instance, even simply catching the ball is composed of opening the hand at the right moment and adjusting the hand to the force of a flying ball. When engaged in an activity, agents carry out a joint combination of many simple physical (and cognitive) actions such as these.

Determining how to act in complex tasks also requires coordinating between multiple parameters in an ongoing and changing activity (Danion and Latash, 2011). A verbal command to play Bach’s first prelude results in a skillful violinist executing a series of cognitive and motor states. An action, however, could be executed in many, possibly incompatible, ways. There are various parameters involved in executing an action: timing, position, velocity, acceleration, joint configuration, etc. To successfully execute an action, individuals are required to approximate an optimal solution from an enormous number of parameters (Latash, 2012).

Consider, again, the action of catching a ball in basketball. The concept of “catch” may correspond to different types of catching actions, such as which body part is needed to execute the movement (e.g., fingers or palm) or its place in a sequence of movements (e.g., “grasp after lifting your arms”). However, the conceptual content does not determine the exact properties of the movement, such as the amount of force that one should exert in grasping an object, or the duration of each pattern of movement constituting an action.

Anti-intellectualists reject the idea that propositional states play a constitutive role in explaining skillful behavior, since they take it that the fine-grained nature of motor states is required for the skillful control of action (Fridland, 2014). Propositional states, according to anti-intellectualists, can only determine movements in a very limited and general way. That is, the content of propositional states cannot specify *all* the elements involved in the execution of movements (see, e.g., Pacherie, 2011).

A central strategy adopted by intellectualists to explain the fine-grained nature of the informational states guiding skillful actions is to appeal to *practical* representations (Pavese, 2017, 2019, 2020). Practical representations allow for propositional content to be sufficiently rich in detail to capture this fine-grained information. A practical representation stands for a method composed of a set of motor commands that translate the individual’s intentions into a set of operations that are carried out by the motor system. Motor tasks are performed according to a method, which specifies a particular pattern of movement based on the various parameters involved in executing an action. Hence, how propositional contents are practically represented is supposed to account for the specific way a skillful action is executed.

An anti-intellectualist, however, can reply that motor representations are *indeterminate* with respect to propositional content attribution since motor representations often exhibit a kind of open-ended structure. Burnston (2020) has recently mounted such a critique against intellectualism. He argues that the representations guiding skillful actions do not have the right structure to be described as propositional knowledge, as they are indeterminate: “there is no fact of the matter about exactly what contents are represented” (ibid, p. 13). He argues that propositional states are too fixed to pick from the many motor patterns available to perform an action, thereby hindering needed flexibility.

The disagreement between intellectualists and anti-intellectualists can be characterized, then, as concerning the informational states that determine how skills are instantiated in action on various occasions. While intellectualists emphasize propositional states as a way to account for the stability of skill across different instantiations, anti-intellectualists stress that, for the purpose of flexible control, these informational states cannot be known beforehand in a fixed and determinate form. These views conflict, therefore, on how to understand the relation between different skill instantiations and the informational state guiding an action.

To advance the dialectic between intellectualists and anti-intellectualists, we propose to reframe this debate in terms of the control of motor *variability*. To explain why a particular action that is controlled by a motor representation occurred, we should cite the content of an intention (Burnston, 2017). By focusing on motor variability, we ask which informational states allow for the *same* action to be executed *differently* given that the content of the intention cannot account for that difference. The problem, plainly, is this: Given the sheer number of potential moves to perform a given action, why does the belief that *x* is a way to catch the ball, for example, lead to the way by which the ball is eventually caught?

In sum, we suggest understanding the puzzle of KH as being about which informational states could guide skillful action while being sensitive to what it requires: context-sensitivity and flexibility. What is at stake in the dialectic between intellectualists and anti-intellectualists, we submit, is how skillful agents get it right despite the high level of motor variability involved in employing a skill across different instantiations. So, the first challenge for views of KH is explaining the relation between motor variability and the informational states guiding skillful action.

## The Importance of Practice for Knowledge-How

The second challenge concerns the role of practice in acquiring KH (Fridland, 2013). Both intellectualists and their opponents would, supposedly, agree that skills are acquired after a specific process—*practice*—has taken place. When an agent only starts to learn how to F, she does not yet *know how* to do it. It is through practice that one comes to know how to F. But practice transforms the nature of the action concerned. Movements become more accurate, fine-tuned, or faster due to practice. The more we practice, the better we become in executing an action.

It is the transformation of action through practice that makes automaticity a major aspect of skillful action. Skillful actions are automatized to ensure successful execution and reduce the cognitive resources required to execute them. A controlled process may involve dealing with stress (“I must score a goal to win the match”), and trying to actively suppress noise, such as a roaring crowd, or to simultaneously perform another unrelated action. But one would not be very efficient in acting, if stress and distraction, for example, interfered continuously with executing a task. Automaticity results in “sealing” the motor process from external influences, thereby guaranteeing its execution.

Crucially, however, automatic actions are not homogeneous (Moors and De Houwer, 2006). Most actions (e.g., driving) may involve components that are both automatic (e.g., shifting manual gears) and controlled (e.g., driving at a green light). Moreover, control may be regained over components of an automatic action. Hence, describing an action as automatic does not entail that it cannot come under one’s control. A central feature of automaticity, thus, involves the minimization of control. An action is typically conceived as automatic when an agent does not possess complete control over its execution, where altering an action can happen at different stages. An action under our complete control may be initiated, altered, and stopped by an agent. An automatic action, by contrast, would be an action that exhibits a lack of control in at least one of these ways (see, e.g., Wu, 2020).

Despite its vital role in skilled action, it is not clear why the automatization of actions following practice would be deemed intelligent. Supposedly, automatic actions do not require any *understanding*. We simply do what we repeatedly trained to do. Knowledge associated with skillful action cannot, accordingly, derive its intelligence from mere mindless repetition. Bernstein (1967)—one of the founders of motor control science—expressed this worry by saying that “practice is a particular type of repetition without repetition, and that motor training, if this position is ignored, is merely mechanical repetition” (p. 134).

The role of automatization and how it transforms an action through practice to make it more successful has been central in the KH debate. We want to know what practice does: “How does practice change our behaviors such that they go from being awkward, unskilled actions to elegant, skilled performances?” (Fridland, 2019, p. 761). Clarifying how this process unfolds is important as practice transforms the informational character of action-guiding states. The puzzle of practice concerns how skillful actions retain their cognitive character following that transformation (Fridland, 2017a).

Intellectualists typically adopt one of two lines of response to account for this transformation. The first line of response invokes the notion of motor chunking. Chunking is a process by which many serial actions are grouped into units of commonly co-occurring actions. Such “chunks” may then be executed as an integrated unit, i.e., a single unified movement pattern, thereby facilitating action execution (Diedrichsen and Kornysheva, 2015). Chunked sequences are treated as a computational structure without parts initiated by a propositional state. Pavese (2019) has argued that chunking is what makes improvement in performance through practice possible, because tasks that had to be executed through different instructions are now executed directly (p. 796).

Relatedly, Haith and Krakauer (2018) advanced the idea of cognitive caching—a fast and efficient memory retrieval process. In this line of response, components of a motor task are transformed, through practice, into frequently occurring computations that are cached for faster retrieval. The underlying assumption is that cognitive overload is reduced by only caching specific computational steps to facilitate action selection. Explicit instructions are transformed through practice to automated responses that reflect the execution of previously learned content (Krakauer, 2019).

Anti-intellectualists claim that automatic processes may be sensitive to intentional content, thereby being cognitively controlled, while denying that motor representations are necessarily responsive to propositional content. According to Fridland (2019), practice structures the motor routines constitutive of skilled action, by integrating and parsing motor sequences through motor chunking. She distinguishes between two complementary processes: concatenation and segmentation. Concatenation is an associative process that integrates motor sequences, whereas segmentation reflects a cognitively controlled process, responsible for parsing motor sequences. The joint operation of these two processes expresses the cognitive character of automated motor sequences.

Intellectualists and anti-intellectualists agree that informational states are transformed through practice but disagree over whether these transformed states are propositional in nature. Interestingly, both views refer to chunking but do not assign this transformative process any epistemic import. What is epistemically assessed is whether the motor sequences involved were cognitively initiated, constituted, or construed. This makes the computational advantage brought by automatization mysterious. Is it simply the grouping together of motor routines that reduces the cognitive burden? Does not the time experts spend practicing make any difference in their knowledge? These questions are left open by intellectualists and anti-intellectualists. The second challenge for views of KH, we submit, is accounting for the *epistemic* value of practice.

## Two Desiderata for Accounts of KH

Above, we have flagged two outstanding challenges in the debate between intellectualists and anti-intellectualists. The first is that explaining skills requires understanding how successful actions are guided by informational states despite various instantiations. The challenge for both views is to account for how informational states provide flexibility by allowing for the same action to be executed differently. The second challenge concerns the transformative function of practice. Practice is highly relevant for explaining skills, but its epistemic significance is unclear. The problem for both intellectualists and anti-intellectualists is to clarify the epistemic role of the transformative function of practice, specifically its close relation to automaticity.

Thus far, the discussion points to what a plausible account of KH should look like. First, the account should clarify the nature of informational states that play a role in controlling skillful action while allowing for variability in the deployment of skillful actions. The difference between different instantiations underlies important aspects of skillful action, specifically, its context-sensitivity and flexibility to task demands. Let us call this the *flexibility* desideratum: the account should explain the *flexible* structure of KH.

Second, a KH account needs to explain the informational transformation that occurs as actions are automatized due to additional practice, and, especially, how this process plays an epistemic role. Importantly, the epistemic profile of how skills are acquired differs from how propositional knowledge is acquired: motor behavior changes with practice and repetition in a way completely distinct from more clear cases of propositional knowledge, e.g., memorization (Fridland, 2013). Practice leads to automatization, thereby reducing the cognitive load associated with complex skillful actions. This process has epistemic import, as it allows flexibility in executing skillful actions. Let us call this the *offload* desideratum: the account should explain the epistemic features of skills gained by automatization. In the next section, we propose a new conceptual framework that avoids the two challenges discussed above, while satisfying the flexibility and offload desiderata.

## A Conceptual Framework: Functional Information

The previous section presented two challenges that make it difficult to adopt intellectualism or anti-intellectualism for explaining KH. First, what informational states determine the required specificity to account for flexible and fine-grained skillful performance? Second, do acquired skills gain an epistemic character through practice? The account presented hereafter is a hybrid one. It is intended to account for these two challenges by satisfying the *flexibility* and *offload* desiderata. A key premise in the proposed view is that KH can be understood as an informational relation, and that only in *some* cases that relation will be genuinely *propositional.*

While the development of a theory of KH is left as a future task, our present aim is rather modest. We aim to spell out (a) what functional information is, and (b) how information plays an explanatory role in understanding KH, while (c) satisfying the flexibility and offload desiderata. In subsection “Functional Information in a Nutshell,” we briefly explain what functional information amounts to. Then, in subsection “A Taxonomy of Functional Information,” we introduce a taxonomy of functional information and clarify how it can play an explanatory role in understanding KH. In the subsequent subsections “Functional Information and Motor Variability” and “Functional Information and Practice,” we argue that an information-based account of KH can straightforwardly satisfy the two desiderata.

### Functional Information in a Nutshell

Different accounts of functional information have recently been offered (e.g., Rathkopf, 2017; Mann, 2018), mainly in biological contexts, but Fresco et al. (2018) offer a possible conceptual framework that may be suitable for explaining KH and will, thus, be the focus of our discussion. “Functional information” here means any difference in the external or internal milieu of a system that has made a systematic, causal difference to the agent’s goal-directed behavior. For a biological structure or process to have a systematic functional effect (a) it should have been produced through phylogenetic and/or ontogenetic selection processes, and (b) there should be a consistent relation between variations in the spatiotemporal form of the input and the corresponding changes in the receiver’s response.

Given that functional information, in this view, is produced through selection processes, a clarification about the nature of selection is in order. Selection is understood here in a broad, Pricean sense to include all the sampling processes that may contribute to adaptation *via* variation and selective retention (Price, 1995). The processes of variation, generation, and selection may be related due to a particular property, which was acquired during learning, leading to a change in the value of this property at the next generation or time-step. Such general notion of selection includes not only the familiar Darwinian type–operating on multiplying, replicating entities, but also sample selection—a process of selecting a subset from a set according to some value criterion without multiplication or replication. Accordingly, even the selection of specific radio stations with the turning of a dial or choosing specific oranges from a set of oranges qualify as cases of sample selection.

Learning plays a central role in this conceptual framework, and we thus briefly elaborate how it should be understood here. It is roughly understood as a selection process that is based on exploration and stabilization processes. Through learning, the receiver’s interpretation system undergoes a stabilization process in response to a relevant environmental condition. In what follows, we will specifically focus on reinforcement learning, a type of learning that is highly relevant to skillful behavior (Fu and Anderson, 2006; Shadmehr and Ahmed, 2020). Roughly speaking, reinforcement learning is learning how to map situations to actions that maximize reward (Sutton and Barto, 2018). As Niv (2009) remarks “computationally, such decision making is treated as attempting to optimize the consequences of actions in terms of some long-term measure of total obtained rewards” (p. 2).

Consider trial-and-error learning in maze navigation as a case in point. A navigator can proceed in many different possible paths from the entry point to the exit point. The initial space of possible paths is large. Any failed *exploration* of a path should, in principle, result in eliminating that specific path from the set of possible paths. Repeated exploration of this space leads to a selective stabilization on a smaller subset of possible paths that *do* lead to the exit point. Suppose that an agent exploits some difference-maker (e.g., a flashing light correlated with an exit point) to facilitate the navigation.^2^ She may exploit more functional information (by observing the flashing light) with the elimination of possible trajectories to the exit point. It can, then, be argued that under specific conditions—on which we do not elaborate here—she acquires KH to successfully navigate the maze by standing in a particular informational relation to *that* maze. This relation consists of three relata: an agent (the navigator), a difference-maker (the flashing light), and some state of affairs (the maze’s exit point).

This case exemplifies a more general observation. Adaptive outcomes are reinforced when they change the organism’s behavioral dispositions. In the case of navigation, successful and unsuccessful exploration of the maze modifies the long-term measure of obtained rewards, thereby leading to the stabilization on a smaller set of potential trajectories through the maze. The learner can extract functional information in virtue of the stabilization process that occurs in response to the visual stimulus, e.g., the flashing light, and is underpinned by the fact that the visual stimulus is highly indicative of the maze’s exit point—the state of affairs acting as a reinforcer. Such a process, we suggest, occurs whenever an agent learns a skill.

### A Taxonomy of Functional Information

To support the claim that KH can be understood as an informational relation, we briefly introduce a plausible taxonomy of functional information, which distinguishes amongst a “datum,” “sign,” “signal,” and “symbol.” A datum/sign/signal/symbol is a difference that makes a potential or actual difference, for example, to the receiver’s chances of locating food, riding a bicycle, or updating the degree of credence. Such a difference makes the receiver respond in a way that it can (actually or potentially) alter the receiver’s state in a (usually) functional manner. Importantly, there is an inclusion relation between the types of aforementioned difference-makers. A symbol is a subtype of a signal, which is a subtype of a sign, which is a subtype of a datum.

A datum is a regular act, event, process or structure to which a receiver can, but does not yet, functionally respond by being sensitive to variations in its spatiotemporal form. A sign is a datum the receiver evolved to either overtly respond to or acquire an altered disposition to respond to through past phylogenetic, ontogenetic, or cultural selection. A signal is a sign that may have a learned component and is sent by a sender that evolved, through past natural, ontogenetic, or cultural selection, to emit it as a sign for particular receiver types.^3^ Finally, a symbol is an intentional signal that is part of a systematic, rule-governed, self-referential system. Symbols are largely believed to be accessible only to humans.

The functional information an agent can exploit through signs, signals, and symbols changes as the agent learns. Many regular features in the world qualify as data for an agent so long as the agent is capable, in principle, of identifying them, and responding to them functionally under the right conditions. For example, despite being correlated with rain, dark clouds do not even qualify as data for a blind person who cannot see them. When a datum upgrades to a sign through a learning process, the agent’s interpretation system undergoes a stabilization process in response to the environmental condition.

Consider how one learns to play guitar. Initially, each guitar string is a *datum* for the novice guitar-player. Strumming any of the strings produces a unique sound depending on the resistance of the string and how it is strummed. Many notes can be played on the guitar in different fingerboard locations using more than one finger. Accordingly, the number of possible ways a musical piece can be played grows exponentially large as the number of music notes increases. The guitar-player begins by learning to play *specific* chords by placing her fingers on the strings at the different frets, leaving some strings open, and strumming some of the strings in a given order. Initially, she is not yet accustomed to the resistance of the strings and how much finger pressure should be exerted as the fingertip touches the string. With practice, she learns to play several chords in a *sequence*. This is the exploration phase during which several data (i.e., specific strings) become signs for that learner. When pressure is applied to the string and then released, the moving string is a difference-maker correlated with some sound pattern in the world.

A similar analysis applies in the case of signals. While a sign may be environmental, a signal originates in a sender that was selected to emit signs for receivers. One way to understand what signals are is to consider how imitation plays a role in skill acquisition. Imitation is a form of learning in which “the observer exhibits a behavior that is topographically similar to the behavior of the demonstrator; the parts of the observer’s body move in the same way, relative to one another, as the parts of the demonstrator’s body” (Heyes, 2012). For example, by observing someone catching a ball, a baby can catch the ball by moving her hands in the same way, thereby imitating that person’s actions.

It is important to keep in mind that signals need not be part of a self-referential rule-governed system such as symbols. This is apparent in imitation when even initially meaningless data can be copied so long as the receiver can regularly and functionally respond to the sign sent by the sender. To understand signals as a distinctive type of functional information, we may consider some specific neuropsychological deficits associated with imitation versus deficits associated with pantomime of tool-use. Consider the case of motor apraxia. Motor apraxia is a neuropsychological motor disorder characterized by the inability to correctly carry out a learned motor act despite the preserved capability of the motor system to produce the intended movement (Heilman and Rothi, 1993). Motor apraxia is not the result of motor-related deficits, e.g., paralysis, but is hypothesized to involve the loss of both symbolic and non-symbolic *information* related to learned movements.

Imitation and pantomime of tool use can be dissociated in the case of apraxia, thereby showing that some cases of learning are based predominately on signals and others on symbols. There are patients with severe problems in imitating gestures, but who can produce pantomimes of tool use (Goldenberg, 2013). For instance, they will have no difficulties demonstrating how to brush their teeth, but they will not be able to imitate the examiner performing the very same teeth brushing movements. This functional separation reflects an informational distinction. In imitating a gesture, the patient has to track the movements of the examiner and convert this non-symbolic information to the relevant movement (Rothi et al., 1997). In pantomime, though, a different process unfolds. The form of communication taking place in pantomime is *intentional* and governed by rules specifying the correct way to perform a symbolic gesture such as using a tool. To demonstrate how to use a hammer through pantomime, the patient should have symbolic information about the function and identity of the tool (Canessa et al., 2008).

One way to understand signals is to focus on the informational relation missing for apraxia patients who cannot imitate. Since the informational relation in imitation is based on the interaction between the sender and the receiver, e.g., “copy how I move my hand” and not on the rules governing the interpretation of the gesture, e.g., “show me how soldiers salute,” imitation is an example of communicating with non-symbolic signals. The fact that an individual may lack this form of communication while retaining a more symbolic form of communication as in pantomime, attests to the distinctive role non-symbolic signals play in skill acquisition. It is the spatiotemporal variations in inputs emitted by the sender that determine which movements are selected by the receiver.

Finally, consider an instruction manual on how to build a table as an example of exploiting symbolic information. A manual consists of a sequence of instructions (i.e., symbolic information). Each instruction specifies, to a varying degree of granularity, the process of performing a given task (e.g., “unbox the entire contents of the box”) with a clear, specific goal (e.g., “to inspect all the items that comprise the table”). However, the manual presupposes the possession of prior knowledge or skills. An instruction may specify the use of a screwdriver (or a hammer) in conjunction with some parts provided in the box (a screw or a nail), but it will not specify *how* the tool is used. A skilled agent (e.g., in using a hammer) should be able to build the table by exploiting the symbolic information assuming that the necessary parts are included, and the instructions are accurately performed in the right order. But as anyone who has tried to build a DIY table recognizes, while these manuals are contoured to fit many scenarios, they will rarely be exact.

Besides, a craftsperson will exploit only the task-relevant information in the manual and ignore all other information (Haider and Frensch, 1996). A novice will assemble the table by trial-and-error and exploration of the symbolic information available to her, particularly when following some instruction does not produce the desired goal. We turn next to argue that a detailed account of KH that is based on functional information can avoid the two challenges discussed above.

### Functional Information and Motor Variability

Recall that the first challenge in the debate between intellectualists and anti-intellectualists concerns how successful actions are guided despite their varying levels of detail. Intellectualists should account for how motor variability might drive flexibility, while anti-intellectualists should explain how such variability is stabilized. In what follows, we argue that an account of KH based on functional information may offer a solution to the challenge of motor variability in a way amenable to both propositional and non-propositional states.

We take it that the capacity to adapt to environmental variation is central to the flexible execution of action in skillful individuals (Levy, 2015). To flexibly execute an action, one needs to employ a skill in unfamiliar situations where the values of known movement parameters are sensitively modified to fit different task requirements. For an individual to apply a judo throw on a new, much taller, opponent, for example, requires a change in limb position. The ability of an individual to change her actions to fit a wider range of variations in task context can, thus, be used to evaluate the level of flexibility a skillful individual possesses.

It is important to recognize that motor behavior naturally involves a high degree of variability. Intuitively, *motor* variability—the variation inherent in patterns of movements, muscle activity, and postures—represents noise and inaccuracy in executing an action. *Movement* variability, however, can also be considered as a way in which agents adapt to differences in task context (Herzfeld and Shadmehr, 2014; Dhawale et al., 2017).^4^ In this view, motor variability plays a similar role to the role of genetic variation in natural selection: a resource that shapes adaptive behavior through selection by functional outcomes.

One line of evidence supporting this interpretation of motor variability concerns tasks that examine the influence of reward history on trial-to-trial motor variability (Wu et al., 2014; Barbado et al., 2017). For example, in a task in which participants were rewarded after reaching a hidden target, it was found that motor variability is modulated by a change in reward contingencies of the recent previous trials (Pekny et al., 2015). Increasing or decreasing reward contingencies made the movement patterns less or more variable, respectively.^5^ These studies suggest that individuals probe the consequences of various motor patterns, a process that allows them to contextually adjust motor output accordingly.

Such tasks point to the role functional information may correspondingly play in modulating variability to flexibly guide motor action. Which subsequent actions are executed depends on which components of an action were first initiated, on environmental circumstances, and, most importantly, on how *successful* previous actions were. If a movement led to unpredicted changes in the agent’s environment, a movement pattern that differs from the one planned might be executed instead. The solution often comes from relying on variations in an informational relation and not from possessing a *determinate* way to F.

The way functional information contributes to skill acquisition and execution is by modulating the receiver’s action space. In our proposal, functional information is produced through a selection process that includes the sampling of movement patterns *via* variation and selective retention. Exploration of the action space—the space of possible patterns of movement—results in eliminating unsuccessful movement patterns. Repeated exploration of this space leads to a selective stabilization. If applying the same judo throw against different opponents results in a successful outcome, fewer motor programs would need to be weighed before selecting the best course of action. As information becomes available to the individual about the functional values of various actions, exploration of this space would decrease.

Importantly, motor actions need not become rigid by selective stabilization. Increasing or reducing motor variability depends on the functional information that the receiver can extract from an informational relation. When the difference-maker is less informative about the outcome of a movement, the relative amount of motor variability increases, reflecting the individual’s exploration for a new optimal point in a wider space of motor parameters. As the motor system stabilizes on action variants associated with functional information, motor variability is reduced. The variability in motor behavior reflects, thus, an adaptive process.

The amount of functional information an agent can extract partly depends on how similar a given context is to the one in which the informational relation was initially stabilized. Consequently, the similarity between task contexts would determine the variability of motor behavior. In more similar contexts, movement patterns would tend to be more stable. In less similar contexts, by contrast, more variation in movement would follow. Hence, the inevitable variability that exists in different learning contexts leads to flexibility in the motor behavior employed no matter how minute, preventing any fixation of motor behavior in general.

Consider again the example of applying a judo throw against different opponents. Increasing or reducing the variability in employing a technique depends on the functional information extracted from informational relations such as the size of one’s opponent as a *sign*, e.g., the amount of force one should exert in grasping one’s opponent. That depends on how similar the given context is to the one in which the informational relation was initially stabilized, e.g., the body weight, height, and strength of one’s typical opponent. Note that, in our proposal, no appeal to propositional knowledge is made in the present example.

Nevertheless, the same process would also occur in cases of signals, such as in learning by imitation, or symbolic information, such as instructions. Consider cases of symbolic information conveyed by verbal instructions. Verbal instructions provide athletes with information on how to perform an action and are used to focus the athletes’ attention on the most relevant features of the context (Wulf, 2013). Verbal instructions may also provide functional information through feedback. Following the execution of a skilled task by an athlete, her coach may provide her feedback to augment the sensorimotor feedback she obtains naturally (Porter et al., 2010). For instance, a coach can tell a judoka “The first step when attempting to apply a judo throw is to focus on the opponent’s legs” to direct her attention to the relevant difference-maker, such as the positioning of the opponent.

Instructions of that sort are based on the information possessed by experts about a given activity. Being exposed to a wide range of similar situations, the relevant informational relations are broadly stabilized in experts. Verbal feedback, from a coach, say, can, thus, shorten the exploration phase of an individual by directing her attention to the specific movements that should be selected and executed. In this way, symbolic information can constrain the space of possible actions and reduce motor variability by relying on individuals for which these informational relations have been stabilized in previous learning contexts. For example, verbal instructions provided by the coach can reduce the range of movements performed by the athlete as those instructions are based on the coach’s experience as a former athlete.

The connection between motor variability and functional information may, therefore, offer a possible solution to the flexibility of skills challenge. The function of motor variability can be understood as a form of flexible adaptation to changing task demands. As argued, functional information plays a central role in modulating such variability. So, the functional information exploited directly supports flexible motor behavior. By changing the level of motor variability exerted to fit the functional information received, individuals are able to produce successful actions in different task contexts.

Note that this adaptive process need not to reflect the employment of propositional knowledge. First, practical representations that qualify as standing knowledge states, are “a *predetermined* set of [motor] commands” (Pavese, 2019, p. 810, italics added). But, if what we have argued thus far is correct, the flexible employment of skillful behavior is *not* predetermined and depends on the trade-off between stability and variability. Specifically, the ability of the motor system to modify its output in order to fit the variation in an informational relation is vital to the flexibility of skillful performance.

Second, the advantage of our proposal over one that trades in *propositional* information exclusively is that it may serve as a bedrock to account for the relation between animal cognition and skill. Some informational links that require a propositional relation are not available to most non-human animals, though it does seem plausible that they *do* possess skills (Springle, 2019). To account for evidence about skills in non-human animals, the explanatory framework should not necessarily commit to the psychological role of propositional content in non-human animals.

Our view also differs from current anti-intellectualist views. According to Burnston (2020), the type of representations processed in skill employment and learning are structured sensory representations (SSR). These representations are:

“multiscale and holistic…learning effects are driven both by particulars, and by correlational and configural relationships that generalize over particulars…The picture that emerges from these properties is that SSRs are multilevel constraints on actions, without being determinate representations of them” (Burnston, 2020, pp. 11–12).

While there are some similarities between our view and Burnston’s, especially the emphasis on capturing “both variance and invariance” (ibid, p. 11), functional information is different from SSR in several aspects. First, although representations can be viewed as a subtype of functional information, not every instance of functional information is a representation (certainly every symbol is a representation; see Fresco et al., 2018, Sect. 6). Second, our proposal allows for both discursive and non-discursive representations to play a role in KH. What alters the way functional information influences skillful behavior are changes in reward history. Motor variability is a non-exclusive example of such a process.

Moreover, while Burnston (2020) focuses on how “general configural patterns intersect with particulars at multiple scales to determine performance” (p. 14), processing functional information involves the modulation of the receiver’s action space such that the information available about the functional values of various actions changes the possible courses of action open to the receiver. Instead of an open-ended structure, we contend that the control structure guiding action is repositionable. The action space does not consist of both general patterns and particulars, as in Burnston’s view, but contracts and expands given the informational relation between the receiver and the difference-maker.

The importance of motor variability indicates that part of what makes skills so flexible is that the information guiding them is sensitive to variations in relations between the skilled individual, the sign/signal/symbol, and the relevant state of affairs (e.g., performing a judo throw on opponents of varying heights and weights). Skillful performance operates through a control structure that is only partly fixed and determined, whereas its indeterminate parts are highly dependent on variation in informational relations. The *flexibility* desideratum, thus, is satisfied by the relation between motor variability and functional information.

### Functional Information and Practice

Our proposal can accommodate the practice-related challenge by appealing to a reduction in the number of computational steps required to perform a specific action that follows the acquisition of functional information. It thereby also satisfies the second desideratum, that is, explaining skillful behavior while accounting for the epistemic features of skills gained by automatization. In this final subsection, we elaborate on the way functional information clarifies the epistemic relation between practice and KH.

As one becomes more skilled in performing an action, an individual gains more functional information from a difference-maker. When one’s skills develop, her exploration space becomes smaller, and she stabilizes on the “right” course(s) of action. In this process, the functional information one gains from a difference-maker increases until it plateaus and reaches its maximum for the agent. In other words, relevant alternatives are eliminated as one gains more action-related information.

Skills develop as specific actions are automatized. Automatization reduces the computational load required to perform a skillful action. Skills are intertwined with the reduction of complexity. As one gains more information from a given difference-maker, one’s uncertainty about the suitable course of action(s) reduces, making an individual more skillful in performing the task. For instance, being skillful in cycling depends on information the individual has from various difference makers, such as the sensitivity of the bicycle handlebars, the balance between the agent’s weight and the bicycle durability, the slope of the terrain, and so on. The individual becomes more confident about how things would turn out, leading, for example, to focusing less attention on balancing herself on the bicycle. Increased proficiency leads to the reduction of uncertainty in relation to such difference makers.

Automatization is the result of reduced uncertainty due to the increased amount of functional information (in the stabilization phase) gained from the difference-maker. Lower uncertainty about the outcome of an action reduces the number of computational steps required for achieving a goal (predictions become more accurate, corresponding actions are faster, etc.). Instead of regularly checking whether one is safely balanced while cycling, this sub-action is unified into a whole action, computed as a single procedural course of action. This enables an individual to perform fewer computational steps, as more information about sub-actions is extracted, and uncertainty about the desired outcome is reduced. Therefore, gaining more information from the difference-maker reduces the number of computational steps required to perform a skillful action, thereby reducing cognitive load.

An informational account of KH, thus, has the potential of explaining the epistemic change that occurs through practice. We strive to minimize the negative impact of noise, and thus to enact specific movements that leave us with less noise, i.e., less *uncertainty* concerning the movement’s outcome. For instance, in basketball, one would tend to pass the ball for long distances, as the outcome of such action is harder to predict in real-time. This may also explain why, in particular cases, expert players choose to perform actions that less skilled players normally would not; they are more warranted about the results of their movements.

The reduction of uncertainty about one’s actions that follows from automatization lends a natural explanation about the flexibility of skilled action. By reducing the uncertainty of the outcome of an action, a skillful individual may engage in cognitively demanding processes as cognitive resources now become available. If, for example, one does not need to regularly check whether one is safely balanced while cycling, one can be more attuned to road conditions, thereby planning an appropriate course of action.^6^ Automatization leads to the flexibility of skilled action through minimizing the control over an action whose outcome we expect to occur.

Thus, an informational account of KH, in contrast to existing intellectualist and anti-intellectualist views, can satisfy the offload desideratum. Situating automated behavior in the context of functional information can explain the epistemic features of skills gained by automatization. By linking automatization to functional information, it becomes clear that automatization plays an epistemic role through the reduction of uncertainty. This epistemic role also accounts for the organizational aspects of automatization (e.g., caching or chunking) for which intellectualists and anti-intellectualists alike argue. Reduced uncertainty about the outcome of an action reduces the number of computational steps, thereby inducing the reorganization of behavior that follows automatization. An informational account of KH can thereby demystify the computational advantage brought by automatization.

## Conclusion

We have suggested a novel, hybrid view of knowledge-how that appeals to the relation between such knowledge and functional information and avoids two main challenges that plague the current debate between Intellectualism and Anti-Intellectualism. The acquisition of knowledge-how involves an agent becoming more and more able to “extract” functional information from relevant difference-makers. Changes in functional information play a central role in modulating motor variability. By focusing on functional information, one can arguably account for *both* propositional and non-propositional information involved in skillful behavior. Flexibility results from a partly indeterminate control structure making use of motor variability to adapt to variation in informational relations. One can also understand the epistemic role of practice and how automatization arises; gaining more functional information from a difference-maker leads to reduced uncertainty, and, thus, to the reduction of the number of computational steps required to enact an activity.

What makes our view a hybrid one? In some cases, on our view, the best explanation for which informational state guides skillful action requires an appeal to propositions—particularly when *symbols* are used to guide actions. In contrast to intellectualists, however, we claim that propositional states would be constitutive of KH only in cases that involve symbolic information. Not all cases of KH require symbolic information. In comparison to anti-intellectualists, while we accept that the informational states guiding skillful behavior are not generally amenable to explanation in terms of propositional knowledge, we do acknowledge the role propositional states can play in many cases of skillful behavior. Knowing how to perform an action can be manifested in various ways, some are more like propositional knowledge, some are less so. These different ways are uniformly accommodated by our proposal.

## Author Contributions

JN was the leading author but both authors listed have made a substantial, direct and intellectual contribution to the work, and approved it for publication.

## Conflict of Interest

The authors declare that the research was conducted in the absence of any commercial or financial relationships that could be construed as a potential conflict of interest.

## Publisher’s Note

All claims expressed in this article are solely those of the authors and do not necessarily represent those of their affiliated organizations, or those of the publisher, the editors and the reviewers. Any product that may be evaluated in this article, or claim that may be made by its manufacturer, is not guaranteed or endorsed by the publisher.

## Footnotes

1 Some anti-intellectualists ascribe to the view that to know how to F amounts to being *able* to successfully do *x* under different circumstances (e.g., Hawley, 2003).

2 Our use of the term is an extension of Bateson’s (1973) definition of a “bit of information” as “a difference which makes a difference” (p. 315). His definition may indicate effectiveness in the sense of causation, but it may also be a normative indication of importance (Schroeder, 2017). We use a causal-functional interpretation of difference-making.

3 Being a subtype of a sign, a signal also implies a learning process in the receiver as well as the sender.

4 In that respect, our proposed framework accords with recent developments in neuroscience that apply reinforcement learning theory to capture the functional significance of motor variability (see, e.g., Dhawale et al., 2017).

5 There is reason to believe that tracking these contingencies in the value of various actions does not reflect cognitive control (see, e.g., Fridland, 2019). In contrast to healthy participants, the ability to increase variability after unsuccessful outcomes in individuals with Parkinson’s disease was impaired. Individuals with Parkinson’s disease are considered to have an impoverished ability in executing motor actions and, thus, their poor performance on this task does not indicate a cognitive control deficit.

6 This proposal extends naturally to symbolic information, too. For instance, if one is given instructions on how to build a table, then one is less uncertain about the proper place for each part of the table. The symbolic information specifies a sequence of steps that one has to carry out to reach the goal, thereby freeing up cognitive resources by highlighting specific actions whose outcome is more certain than others. However, symbolic information might be insensitive to changes among learning contexts. For a manual to be effective in guiding how to build a table it must ignore the specific details of the user (e.g., being a novice table builder) and particular state of affairs in which that information would be exploited. Hence, symbolic information would reduce uncertainty only in relatively similar contexts.
